# Methicillin-resistant *Staphylococcus aureus* in Saudi Arabia: genomic evidence of recent clonal expansion and plasmid-driven resistance dissemination

**DOI:** 10.3389/fmicb.2025.1602985

**Published:** 2025-06-13

**Authors:** Ahmed Yousef Alhejaili, Ge Zhou, Heba Halawa, Jiayi Huang, Omniya Fallatah, Raneem Hirayban, Sara Iftikhar, Abrar AlAsmari, Mathew Milner, Manuel Banzhaf, Albandari A. Alzaidi, Ahmad A. Rajeh, Maram Abdulmohsen Al-Otaiby, Sarah S. Alabbad, Doua Bukhari, Abdullah N. Aljurayyan, Alanoud T. Aljasham, Zeyad A. Alzeyadi, Sulaiman M. Alajel, Rawan Hamdan Alanazi, Majed Alghoribi, Mashal M. Almutairi, Arnab Pain, Abiola Senok, Danesh Moradigaravand, Waleed Al Salem

**Affiliations:** ^1^Ministry of Health, Riyadh, Saudi Arabia; ^2^Newcastle University Biosciences Institute, Faculty of Medical Sciences, Newcastle University, Newcastle upon Tyne, United Kingdom; ^3^Laboratory of Infectious Disease Epidemiology, KAUST Center of Excellence for Smart Health and Biological and Environmental Science and Engineering (BESE) Division, King Abdullah University of Science and Technology (KAUST), Thuwal, Saudi Arabia; ^4^Faculty of Medical Sciences, Biosciences Institute, Newcastle University, Newcastle upon Tyne, United Kingdom; ^5^Department of Clinical Laboratory Sciences, College of Applied Medical Sciences, King Saud University, Riyadh, Saudi Arabia; ^6^Department of Clinical Laboratory Sciences, College of Applied Medical Sciences, Shaqra University, Shaqra, Saudi Arabia; ^7^Executive Department of Reference Laboratories, Research and Laboratories Sector, Saudi Food and Drug Authority, Riyadh, Saudi Arabia; ^8^Ministry of Environment Water and Agriculture, Riyadh, Saudi Arabia; ^9^Department of Pharmacology and Toxicology, College of Pharmacy, King Saud University, Riyadh, Saudi Arabia; ^10^Pathogen Genomics Laboratory, KAUST Center of Excellence for Smart Health and Biological and Environmental Sciences and Engineering, King Abdullah University of Science and Technology, Thuwal, Saudi Arabia; ^11^International Institute for Zoonosis Control, Hokkaido University, Sapporo, Japan; ^12^College of Medicine, Mohammed Bin Rashid University of Medicine and Health Sciences, Dubai, United Arab Emirates; ^13^School of Dentistry, Cardiff University, Cardiff, United Kingdom

**Keywords:** MRSA, MSSA, bloodstream infection, ST, precision epidemiology, Kingdom of Saudi Arabia

## Abstract

**Objectives:**

*Staphylococcus aureus* is a leading cause of hospital-acquired infections worldwide. Over recent decades, methicillin-resistant *Staphylococcus aureus* (MRSA), which is resistant to multiple antimicrobials, has emerged as a significant pathogenic strain in both hospital and community settings. The rapid emergence and dissemination of MRSA clones are driven by a dynamic and evolving population, spreading swiftly across regions on epidemiological time scales. Despite the vast geographical expanse and diverse demographics of the Kingdom of Saudi Arabia and the broader West Asia region, the population diversity of MRSA in hospitals in these areas remains underexplored.

**Methods:**

We conducted a large-scale genomic analysis of a systematic *Staphylococcus aureus* collection obtained from 34 hospitals across all provinces of KSA, from diverse body sites between 2022 and 2024. The dataset comprised 581 MRSA and 31 methicillin-susceptible *Staphylococcus aureus* (MSSA) isolates, all subjected to whole-genome sequencing. A combination of phylogenetic and population genomics approaches was utilized to analyze the genomic data. Hybrid sequencing approach was employed to retrieve the complete plasmid content.

**Results:**

The population displayed remarkable diversity, comprising 48 distinct sequence types (STs), with the majority harboring community-associated SCC*mec* loci (types IVa, V/VII, and VI). Virulence factors associated with community-acquired MRSA (CA-MRSA), including Panton-Valentine Leukocidin (PVL) genes, were identified in 12 distinct STs. Dominant clones, including ST8-t008 (USA300), ST88-t690, ST672-t3841, ST6-t304, and ST5-t311, were associated with infections at various body sites and were widely disseminated across the country. Linezolid and vancomycin resistance were mediated by *cfr*-carrying plasmids and mutations in the *vraR* gene (involved in cell-wall stress response) and the *murF* gene (involved in peptidoglycan biosynthesis) in five isolates, respectively. Phylodynamic analysis revealed rapid expansion of the dominant clones, with their emergence estimated to have occurred 10–20 years ago. Plasmidome analysis uncovered a diverse repertoire of *blaZ*-containing plasmids and the sharing of *erm(C)*-encoding plasmids among major clades. The acquisition of plasmids coincided with clonal expansion.

**Conclusions:**

Our results highlight the recent concurrent expansion and geographical dissemination of CA-MRSA clones across hospitals. These findings also underscore the interplay between clonal spread and horizontal gene transfer in shaping the resistance landscape of MRSA.

## Introduction

*Staphylococcus aureus* is a major human pathogen responsible for a wide spectrum of infections in hospital settings worldwide. Its clinical significance has been amplified by the emergence of pandemic clones, driven by the acquisition of resistance genes under the selective pressure of antimicrobial use in healthcare environments. Notably, in response to methicillin use over recent decades, methicillin-resistant *S. aureus* (MRSA) has emerged, characterized by the acquisition of the *mecA* gene within the staphylococcal cassette chromosome *mec* (SCC*mec*) (Lakhundi and Zhang, 2018). Globally circulating MRSA clones impose a substantial burden on healthcare systems, contributing to increased mortality, prolonged hospital stays (Inagaki et al., 2019), and significantly higher medical costs and resource utilization (Shoji et al., 2022; Kavanagh, 2019).

The molecular epidemiology of MRSA has been extensively studied globally, utilizing various typing methods. These include SCC*mec* typing, which classifies isolates based on the essential components of the *mec* complex, *spa* typing, and sequence type (ST) as defined by multilocus sequence typing (MLST). Clinical MRSA isolates from humans are predominantly categorized into SCC*mec* types I through V and assigned to clonal complexes (CCs), including CC1, CC5, CC8, CC22, CC30, among others (Lee et al., 2018). The epidemiology of MRSA has evolved significantly, moving beyond the traditional distinction between hospital-associated MRSA (HA-MRSA) and community-associated MRSA (CA-MRSA) (Planet, 2017; David and Daum, 2010). Initially identified in healthcare settings and patients with co-morbidities, HA-MRSA was characterized by multidrug resistance and SCC*mec* types I, II, or III, while CA-MRSA, emerging in the late 1990s, affected healthy individuals without healthcare exposure and typically carried the smaller SCC*mec* types IV, V, or VII, retaining susceptibility to non-β-lactam antimicrobials. However, recent studies indicate that CA-MRSA lineages have increasingly infiltrated healthcare settings, often surpassing HA-MRSA as causes of nosocomial infections in many regions (Bal et al., 2016). This molecular epidemiological shift reflects the adaptability of CA-MRSA lineages, which combine virulence with enhanced transmissibility (Otto, 2013).

The available data on the incidence, molecular characteristics, and mortality rates of MRSA in the Middle East highlight a significant burden in the region (Yousef et al., 2013; Tabaja et al., 2021). Studies from countries such as Kuwait (Alfouzan et al., 2017; Boswihi et al., 2016), the United Arab Emirates (UAE) (Sonnevend et al., 2012), Saudi Arabia (Alkharsah et al., 2018; Alreshidi et al., 2013), and Qatar (El-Mahdy et al., 2014) reveal wide clonal diversity of MRSA. As a major member of the Gulf Cooperation Council (GCC), the Kingdom of Saudi Arabia (KSA) spans a vast geographical area and is characterized by a highly dynamic and diverse population, influenced by substantial immigration and religious tourism. A previous meta-analysis demonstrated a high prevalence of MRSA in KSA, particularly in the western region (Adam and Abomughaid, 2018). Molecular studies from KSA and neighboring GCC countries (Kuwait, UAE, Oman, and Qatar) have identified a wide range of MRSA sequence types (STs). ST5, a globally significant lineage, is frequently reported in the region, often associated with *spa* types t688 and t002 and varying levels of Panton-Valentine leukocidin (PVL) genes positivity. ST8 MRSA, commonly linked to the USA300 clone, has been detected in Saudi Arabia and adjacent countries, typically PVL genes -positive and associated with *spa* type t008. ST22, a predominant lineage in healthcare settings, is also widely reported in KSA, Kuwait and UAE, displaying considerable *spa* type diversity. Other lineages, including ST6, ST80, ST88, ST30, ST1, and ST97, have also been documented, exhibiting distinct genetic traits such as differences in *spa* types and PVL profiles (Alkharsah et al., 2018; Alreshidi et al., 2013). While these studies indicate the growing prevalence of CA-MRSA clones in KSA, they have primarily relied on traditional molecular techniques, which lack the resolution of whole-genome sequencing (WGS). This limitation hampers direct comparisons and accurate lineage tracking due to inconsistencies in reporting. Moreover, most studies have been restricted to individual hospitals or regions, leaving the national population diversity of MRSA largely unexplored. These gaps underscore the need for a comprehensive, WGS-based investigation to provide a more detailed and precise understanding of the molecular epidemiology of MRSA in KSA.

To address this gap, we conducted a nationwide genomic epidemiology study on a systematically collected, large-scale dataset of *S. aureus*, predominantly composed of MRSA strains, from 34 Ministry of Health (MOH) hospitals across Saudi Arabia. Leveraging whole-genome sequencing with both short- and long-read technologies, we attained high-resolution characterization of MRSA population diversity and explored the genomic determinants of antimicrobial resistance and virulence. Our findings represent the first comprehensive genomic analysis of MRSA epidemiology in Saudi Arabia, revealing the expansion and ongoing evolution of CA-MRSA clones. These evolutionary processes are tightly linked to the concurrent acquisition of plasmids and key virulence genes, emphasizing the interplay between clonal adaptation and horizontal gene transfer.

## Methods

### Sampling and collection

This study received ethical approval from the Institutional Review Board (IRB) of King Abdullah University for Science and Technology (approval number 23IBEC027) and IRB of Saudi Ministry of Health (approval number: 23-23 M). As a component of a national surveillance initiative, the reference laboratory at the Saudi Ministry of Health collected samples from hospitals affiliated with the Ministry through standard hospital protocols between January 2022 and 2024. To ensure data integrity, isolates were deduplicated, with only one sample included per patient. A total of 36,286 isolates were identified as *S. aureus*, of which 98% were confirmed to be MRSA.

### Strain identification and susceptibility testing

Identification and antimicrobial susceptibility testing were conducted using the Vitek2^®^ system (bioMerieux, Marcy-l'Etoile, France) in accordance with manufacture provided protocol.

For susceptibility testing, the AST-GP67 cards with a panel of a range of antimicrobials was used. Among the β-lactams, the panel included cefoxitin (FOX), benzylpenicillin (PEN), ampicillin (AMP), amoxicillin/clavulanic acid (AMC), ampicillin/sulbactam (SAM), piperacillin (PIP), piperacillin/tazobactam (PTZ), and oxacillin (OXA). Cephalosporins tested were cefaclor (CEC), cefixime (CFM), cefotaxime (CTX), ceftazidime (CAZ), and cefepime (FEP). Carbapenems included imipenem (IMP) and meropenem (MEM). Aminoglycosides assessed were gentamicin (GEN) and streptomycin (STR). Fluoroquinolones included ciprofloxacin (CIP), levofloxacin (LVX), moxifloxacin (MXF), and ofloxacin (OFX). Macrolides and lincosamides were represented by azithromycin (AZM), clarithromycin (CLR), erythromycin (ERY), and clindamycin (CLI). Other tested antimicrobials included quinupristin/dalfopristin (QDA), linezolid (LNZ), vancomycin (VAN), tetracycline (TET), tigecycline (TGC), nitrofurantoin (NIT), rifampicin (RIF), and trimethoprim/sulfamethoxazole (TMP/SMX). Phenotypic resistance was defined according to the Clinical and Laboratory Standards Institute (CLSI) breakpoints based on minimum inhibitory concentration (MIC) values (Petit and Read, 2020).

### Sequencing, assembly, mapping, and GWAS analysis

Isolates were cultured overnight at 37°C in Luria–Bertani (LB) broth. Genomic DNA (gDNA) was extracted using the DNeasy Blood and Tissue Kit following the manufacturer's protocol (QIAGEN, Hilden, Germany). The quality of the extracted gDNA was assessed using a DS-11 DNA spectrophotometer (Denovix, US), while its quantity was measured using a fluorometric method with a Qubit 4.0 fluorometer and a high-sensitivity double-stranded DNA assay kit (Thermo Fisher Scientific, US). Genomic libraries were prepared for 612 isolates using the MGIEasy Fast FS DNA Library Prep Kit, adhering to the manufacturer's instructions (MGI Technology, China). Enzymatic fragmentation was employed during library preparation, followed by library denaturation and circularization. Whole-genome sequencing (WGS) was performed on the DNBSEQ-G400 platform (MGI Technology, China) using a 2 × 150 bp paired-end read protocol. To prepare sequenced libraries for long-read sequencing, we employed 96-plex Rapid Barcoding Kits for multiplexing. These libraries were then loaded into PromethION flow cells (Oxford Nanopore Technologies) and subjected to a 72-h run following the manufacturer's protocol.

The short reads underwent quality control using the FastQC package in R (v0.1.3). Genomes were assembled using the Unicycler *de novo* assembly pipeline (v0.5.0) (https://www.github.com/rrwick/Unicycler) with default settings (Wick et al., 2017). Genomes were profiled and characterized using Bactopia (v3.1.0) (https://www.bactopia.github.io) to determine the sequence types, SCC*mec* types, and spa types (Petit and Read, 2020). We excluded one genome due to poor assembly statistics. We also used AMRFinderPlus (Feldgarden et al., 2021) pipeline, with identity coverage cut-off values of 50%, for identifying resistance genes.

We annotated the *de novo* assemblies using Prokka (v1.14.5) (Bal et al., 2016) and utilized Panaroo for pangenome reconstruction (Otto, 2013). For the phylogenetic analysis, we aligned the short-read sequences to the reference genome of *S. aureus* NCTC 8325 (accession number: PRJNA57795), employing the Snippy pipeline (available at https://github.com/tseemann/snippy) with its default parameters. We calculated pairwise SNP distances from the core genome alignments. To assess genetic diversity within each province, we determined the average SNP distance by calculating the mean number of SNP differences between all pairs of isolates from the same province. To identify the mutations in the PVL genes of *lukS* and *lukF*, we mapped the short reads against the ST8 *S. aureus* subsp. MW2 (NC_003923) reference genome and used Snippy to identify SNPs. We reported the missence SNPs in these genes. To assess the distribution of SCC*mec* element sizes across the major clones, we used the sizes of SCC*mec* elements that were matched or closely matched (defined as < 10 SNPs) to entries in the SCC*mec* element database in Bactopia. If multiple SCC*mec* types were assigned to a genome, we calculated the average size of the assigned elements. We identified Arginine Catabolic Mobile Element (ACME) genes and, based on pan-genome data, classified them into four groups: ACME-I (presence of *arc* operon genes—*arcR, arcB, arcC1, arcA, arcC2*, and *speG*), ACME-II (presence of *speG* only), ACME-III (presence of *arc* genes only), and ACME-negative (absence of both *arc* and *speG*).

We contextualized our isolates using the Pathogen Detection database (https://www.ncbi.nlm.nih.gov/pathogens/). We retrieved epidemiological SNP clusters on 06/10/2024, from the database, which included genomes with pairwise SNP distances of up to 50 SNPs. The clustering was performed by the Pathogen Detection portal's automated high-throughput pipeline (https://www.ncbi.nlm.nih.gov/pathogens/pathogens_help/#references).

We assessed the significance of associations between accessory genes, SNPs, and resistance phenotypes while accounting for population structure using Scoary (v1.6.16) (Brynildsrud et al., 2016). This analysis was based on the Panaroo output for accessory genes and SNPs identified through post-read mapping to the reference genome. We specifically evaluated pairwise *p-*values (both worst and best) to be smaller than 0.05. These *p*-vlaues are adjusted to account for the confounding effects of population structure, such as lineage effects.

### Transmission analysis and phylodynamic analysis

For the most prevalent *spa* types within the largest sequence type (ST) clones—ST5, ST8, ST80, ST88, and ST672—we performed phylodynamic analyses to estimate key epidemiological parameters for each clone. These clones were ST8-t008, ST88-t690, ST672-t3841, ST6-t304, and ST5-t311. To enhance our dataset and improve the temporal signal, we integrated our genomes with those sequenced in a single hospital study in Jeddah (Sharif et al., 2024).

We selected the isolates with the best assembly metrics, specifically the highest N50, for each clone. The contigs of these selected strains were merged to create local reference genomes. Short reads from each strain within the clone were aligned to these reference genomes to generate a core genome SNP alignment. This alignment was processed using Gubbins (v3.3.1), with five iterations to remove hypervariable regions.

To infer the ancestral origins of the main clones within the SNP clusters, we conducted phylogeographic diffusion analysis in discrete space using BEAST (v2) (Bouckaert et al., 2019). The city of isolation served as the discrete state for each taxon, and we applied a constant population size model with uniform priors on the clock rate. Moreover, a symmetric model with a uniform prior distribution was used for the discrete trait substitution model to analyze the spatial diffusion of the clones. We evaluated the convergence of the Markov chain Monte Carlo (MCMC) chains by ensuring that the effective sample size (ESS) for critical parameters was >150.

To investigate changes in population size over time, we applied a non-parametric growth model to sample population sizes along the dated phylogenetic tree for the five clones. This analysis was performed using the skygrowth.mcmc function, and the results were visualized with the plot function within the Skygrowth package (v0.3.1) (Volz and Didelot, 2018). For the ST8-t008 clone, we reported the age and population growth for the clade containing the PVL genes as well. For the ST6-t304, we exlcuded two divergent genomes.

To validate the results from the phylodynamic analysis, we reconstructed genealogical networks of the sampled sequences, assuming co-sampling of ancestors and descendants for the five clones. This was attained using the adegenet package in R (v1.3-1) (Jombart and Ahmed, 2011), which optimizes the likelihood of the networks based on pairwise SNP distances from core genomes and the corresponding isolation dates. SNP distances were calculated from the BEAST input alignments, where hypervariable sites were excluded. These distance metrics, along with the collection dates, were input into the seqTrack function of the adegenet package. To define genetic relatedness, we applied a 22-SNP cut-off, a threshold previously demonstrated to capture 95% of epidemiologically linked cases within 6 months within hospital settings (Coll et al., 2020). The resulting genealogical networks were visualized using the igraph library in R (Moradigarav et al., 2017).

### Plasmidome analysis

We conducted third-generation sequencing on selected representative samples to characterize the antimicrobial resistance (AMR)-linked plasmids and improve typing within the collection. Forty isolates with the highest number of resistance genes were chosen, representing diverse clades and distinct resistance patterns. Moreover, long-read sequencing was performed on seven untypable isolates, and their profiles were submitted to the PubMLST database (https://www.pubmlst.org) to obtain new sequence type (ST) codes. The list of the isolates subjected to long-read sequencing is provided in Supplemental Table S1.

For library preparation, we utilized 96-plex Rapid Barcoding Kits for multiplexing, loading the libraries onto PromethION flow cells (Oxford Nanopore Technologies) for a 72-h sequencing run, following the manufacturer's instructions. Hybrid assemblies were generated using Unicycler with the conservative option. The resulting contigs were screened for full copies of origins of replication, virulence factor genes, and AMR genes using BLAST, referencing relevant databases. Visualization and validation of assembled genomes were performed using Bandage (v0.9.0) (Moradigarav et al., 2017).

We extracted plasmid fragments containing resistance genes, which were then visualized and annotated using the built-in tools of the Proksee portal (https://www.proksee.ca) (Yu et al., 2018). Resistance genes were identified through BLAST searches on the AMRFinderPlus (Feldgarden et al., 2021) database. To find clusters within *blaZ*-containing plasmids, we conducted blast search and identified plasmids with identical plasmid replicons. To confirm the presence of these plasmids in other isolates not selected for long-read sequencing, we mapped the short reads of these strains against the extracted plasmid fragments, with mapping coverage exceeding 90% serving as confirmation. We visulized phylogenetic trees, along with antimicrobial resistance and virulence factor genes and plasmids with the ggtree package (v3.8.2) in R (Yu et al., 2018).

### Statistical significance tests

We conducted statistical significance tests using R. A one-way proportion test was employed to evaluate differences in ratios, while the one-way Wilcoxon signed-rank test was used to assess differences between means. For parameters inferred from the Bayesian analysis, significance was determined by examining the 95% credible intervals (highest posterior density [HPD]), representing the shortest interval encompassing 95% of the probability density. We analyzed the genetic diversity and incidence of MRSA in each province in relation to population size, using demographic data from https://www.citypopulation.de/en/saudiarabia/cities/, retrieved on 05/06/2024.

### Data availability

Genomic data collected in this study were deposited in the European Nucleotide Archive (ENA) under the study accession number PRJEB71150. The assemblies were uploaded to the NCBI GenBank database under the accession number PRJNA1050907. Detailed metadata associated with the genomes are available in Supplementary Table S1. All the intermediate files and codes are provided in the GitHub directory for the project: https://www.github.com/gzhoubioinf/MOH_MRSA.

## Results

### Overview of the MRSA and MSSA prevalence across the KSA

We conducted a genomic survey of 612 *S. aureus* isolates recovered from different body sites across 34 hospitals in a nationwide hospital network. The prevalence of *S. aureus* exhibited regional variability across provinces; however, no significant correlation was observed between incidence of *S. aureus* and the number of inhabitants of each province (*p*-value from Spearman's rank correlation test >0.05) (see Discussion). Out of the total isolates, 31 lacked the *mecA* gene and were therefore classified as MSSA (methicillin-susceptible *S. aureus*). These figures made our collection representative of 1.5% and 4% of the total 35,560 MRSA and 726 MSSA isolates, respectively. The frequency of MSSA and MRSA was not significantly different in wound (16% MRSA vs. 27% MSSA) and blood (66% MRSA vs. 44% MSSA) (*p*-value > 0.01 from proportion test), showing that both strain types could reside in similar sites. Isolates were selected to maximize geographical and temporal representation.

### High population diversity with dominant clones showing body site-specific preferences

**Figure 1:**
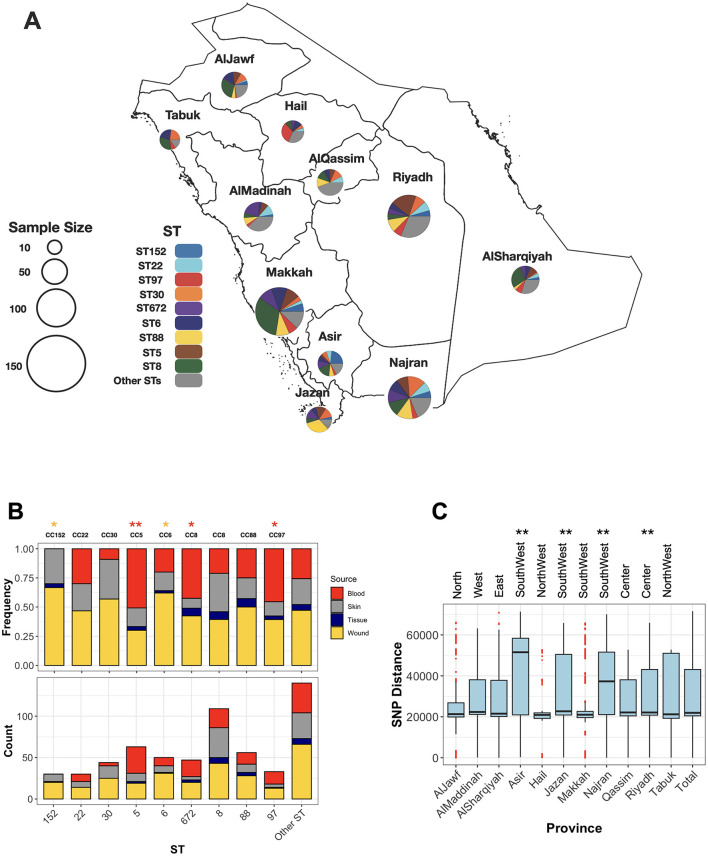
The distribution of major ST clones (STs with more than five representative isolates) across sources and provinces. **(A)** Each pie chart represents the frequencies of major STs in the provinces of the KSA, with the chart size corresponding to the collection size from each province. **(B)** The relative (top) and absolute (bottom) frequencies of STs across body sites of isolation for major STs. Asterisks (*) and (**) indicate significance levels of < 0.05 and < 0.01, respectively, based on a one-sided proportion test comparing the frequencies of blood (red) and wound (yellow) isolates. **(C)** Population diversity of the collection across provinces. Each boxplot represents pairwise SNP distances between genomes within a province. The ** symbol denotes a significance level of < 0.01 based on a one-sided Wilcoxon rank test, comparing the SNP mean distances of each province to those of the other provinces.

The collection was diverse, comprising 48 distinct sequence types (STs). Of these, nine STs were novel, for which new ST identifiers were obtained (Supplementary Table S1). Despite the high diversity, a few ST clones dominated the collection, including ST8 (*n* = 110), ST5 (*n* = 63), ST88 (*n* = 56), ST6 (*n* = 52), ST672 (*n* = 47), ST30 (*n* = 46), ST97 (*n* = 35), ST22 (*n* = 30), and ST152 (*n* = 30), each representing at least 5% of the population (Figure 1A). Isolates from these clones collectively accounted for 75% of the population. MSSA isolates were found in ST30 (*n* = 2), ST5 (*n* = 1), ST672 (*n* = 3), and ST8 (*n* = 1), while the remaining isolates were distributed among other STs. Six STs consisted exclusively of MSSA isolates (ST2867, ST291, ST45, ST7565, ST15, and ST1290). All prevalent STs were isolated from both wound and blood samples, except for ST152, which was not found in blood (Figure 1B). Despite the broad distribution across body sites, some clones exhibited strong associations with specific infection sites: ST5, ST97, and ST8 were more frequently associated with blood, while ST6 and ST152 were more commonly associated with wounds.

### Widespread distribution and regional diversity of MRSA strains

The geographical distribution of different sequence types (STs) reveals that dominant clones are widely dispersed across the country. ST8 and ST6 isolates were identified in all provinces, while other clones—ST97, ST152, ST5, ST672, ST88, and ST97—were detected in most regions, specifically nine out of the eleven provinces (Figure 1A). Population diversity, based on SNP distance distribution, further highlights the broad genomic variation across provinces. The core genome population diversity, measured as the average pairwise SNP distances for isolates within the same province, exhibited a distribution comparable to the nationwide pattern (Figure 1C). Despite this overall similarity, regions in the central province of Riyadh and the southwestern part of the country displayed significantly higher diversity (*p*-value < 0.001, one-sided Wilcoxon rank test). These provinces, characterized by larger human populations and more diverse demographics, may facilitate the introduction and circulation of a wider variety of MRSA strains.

### Diverse antimicrobial resistance profiles and genetic determinants among MRSA clones

**Figure 2:**
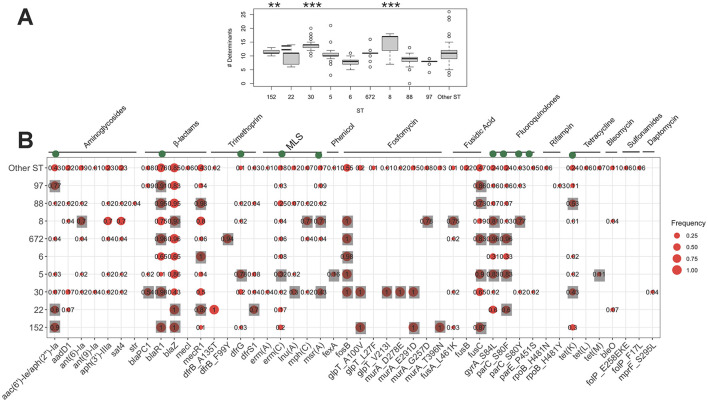
The frequency of resistance determinants (antimicrobial resistance genes/mutations) counts across the major STs. Each boxplot in **(A)** denotes the count of resistance determinants identified by AMRFinder. The ** and *** signs in **(A)** corresponds to the significance levels of < 0.01 and < 0.001, respectively, from the one-sided proportion test, indicating whether the mean of the frequency of resistance determinants count were higher in the ST clone compared to the rest of the collection. The green circles on top genes in **(B)** show the determinants significantly linked with the resistance phenotype from the GWAS analysis. The gray squares in **(B)** shows genes/mutations which had a significantly higher frequency in each ST compared to the rest of the collection (*p*-value < 0.01 from one-sided proportion test).

While the collection was broadly resistant to β-lactams, variation in resistance levels was observed for other antimicrobial classes. ST8 and ST30 clones were significantly more resistant to macrolides (*p-*value from proportion test < 0.01). ST8, along with ST5 and ST772, showed higher resistance to ciprofloxacin (Supplementary Figure S1). ST5 and ST22 isolates were more resistant to trimethoprim, while ST97 and ST152 were frequently resistant to aminoglycosides. Resistance to last-resort antimicrobials, vancomycin and linezolid, was observed in three ST8 and ST88 isolates, and in two isolates of ST5 and ST834, respectively. The resistome analysis further revealed a variable distribution of antimicrobial resistance determinants, with ST8, ST30, and ST152 harboring a greater number of resistance determinants compared to the rest of the population (*p*-value < 0.001, one-sided Wilcoxon rank test) (Figure 2A). Out of 47 antimicrobial resistance genes/mutations, 27 determinants were more frequent in at least one sequence type (ST) (Figure 2B). Several of these resistance genes/SNPs demonstrated strong associations with resistance phenotypes after adjusting for population structure (*p*-value from GWAS analysis < 0.01). These included the bifunctional *aac(6')/aph(2”)* aminoglycoside phosphotransferase, which confers resistance to aminoglycosides and was prevalent in ST97 and ST152; *msr(A)*, an Msr family ABC-F type ribosomal protection protein, and *ermC*, a macrolide-lincosamide-streptogramin B resistance protein, both of which were prevalent in ST5, ST8, and ST30 and confer resistance to macrolides; the trimethoprim-resistant dihydrofolate reductase *dfrG*, prevalent in ST5, which confers resistance to trimethoprim; and the putative tetracycline resistance pump *tetK*, underlying tetracycline resistance in ST88 and ST30 (Figure 2B). Genomic context analysis from long-read squencing data confirmed plasmid contexts for these genes, except for *dfrG*. On the SNP level, distinct evolutionary trajectories for ciprofloxacin resistance in the the quinolone resistance-determining regions (QRDRs) of DNA gyrase and topoisomerase IV were observed across lineages. These included non-synonymous mutations such as S80F in DNA topoisomerase IV subunit A (*parC*), prevalent in ST22, ST5, and ST672, and S84L in DNA gyrase subunit A (*gyrA*), also prevalent in ST22, ST5, and ST672 (Figure 2B). Linozolid resistant isolates haboured the *cfr* gene which encodes a 23S rRNA methyltransferase. The gene was situated on a a conjugative 38Kb plasmid that also carried the efflux pump protein *fexA*, conferring resistance to florfenicol (Supplementary Figure S2). The conjugative plasmid showed >90% sequence identity to the first report of the *cfr*-*fexA* plasmid (accession KC206006) recovered from clinical strains in the US (Mendes et al., 2008). Although no *van* genes were detected, we identified potential missense resistance mutations, which could contribute to intermediate levels of vancomycin resistance. This included mutations in the *vraA* gene (R121I, D59E), which is part of the cell-wall stress VraSR two-component regulatory system, as well as in the peptidoglycan biosynthesis *murF* gene (S350G, T91M, and T301I) (Avison et al., 2002; Cong et al., 2020). Moreover, we did not identify any copy of the *mecC* gene, the emerging homolog of *mecA*, in the entire collection.

### Distinctive virulence profiles distinguish CA-MRSA clones

The screening of virulence genes revealed a higher count in the ST8, ST5, and ST22 clones compared to the rest of the collection (Supplementary Figure S3A). Of the 45 virulence factor genes identified, 34 showed variable presence across clones (*p*-value < 0.05, Fisher's exact test) (Supplementary Figure S3B). Distinct patterns emerged, encompassing toxins, adhesins, superantigens, and enzymes. The *cna* collagen-binding adhesin gene, which aids *S. aureus* in adhering to host tissues, was overrepresented in wound-associated ST152 and ST6 clones, although the role was also shown in bloodstream infections (Iwata et al., 2020). Twenty-four of the 29 superantigen genes, known for producing potent immunostimulatory exotoxins linked to bloodstream infections (Spaulding et al., 2013; Maeda et al., 2016), were variably present but overrepresented in at least one major clone compared to minor clones (Supplementary Figure S3B). These superantigen genes contributed to the higher virulence gene count in bloodstream-associated ST5 and ST8 clones. The superantigen toxic shock syndrome toxin-1 protein (TSST-1), linked to toxic shock syndrome in *S. aureus* infections, was detected in 43 out of 612 isolates (7%) across eight sequence types. The gene showed significant prevalence in ST22 strains and was sporadically present in ST5, ST672, and ST8 clones. A hallmark of CA-MRSA infections, the pore-forming cytotoxin Panton-Valentine Leukocidin (PVL) genes (*lukSF-PV*), which target white blood cells, were present in 42% (258/612) of genomes across 21 of the 45 STs, including 4 novel STs we reported. This locus was found in all major clones except ST97. The majority of ST152, ST30, and some lineages of ST88, ST30, and ST22 carried these genes, although their frequency was lower in the ST5, ST672, and ST6 clones (Supplementary Figure S3B). The analysis of polymorphisms associated with the PVL genes identified four missense mutations—two in *lukF* and two in *lukS* (Supplementary Table S2). The mutations Tyr30Phe and Glu322Lys were found exclusively in ST8 and ST30 isolates and appeared to be lineage specific, whereas the SNPs in *lukS*, including Arg176His and Phe157Tyr, were present across multiple lineages. These polymorphisms have been previously reported in different clones (David and Daum, 2010). However, their impact on leukotoxicity remains unclear and may represent neutral, lineage-associated polymorphisms (Bal et al., 2016). Another key virulence factor of CA-MRSA, ACME-I—previously identified as a contributor to the USA300 clone—was restricted to ST8, which also harbored PVL genes and comprised 84 isolates. In addition to ST8, ACME-I was detected in two less common CC8 sequence types: ST7435 (2/2) and the novel ST9384 (1/1).

### Clones with smaller SCC*mec* elements and quinolone resistance-determining regions harbored higher virulence and resistance gene content

**Figure 3:**
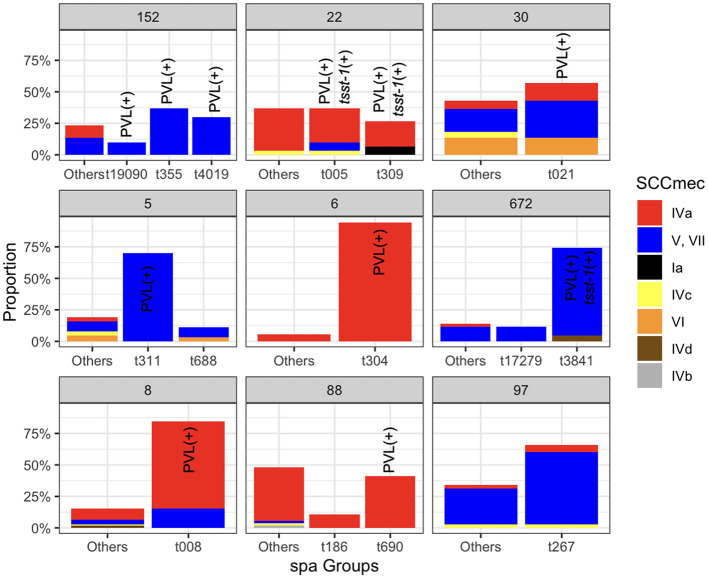
The relative frequencies of the spa types SCC*mec* types across the major STs. Spa types with a relative frequency of < 0.1 for each clone were aggregated. Bars corresponding to the sub-clones that have at least one isolate carrying the PVL and *tsst-1* genes are marked.

Each clone in the population exhibited diverse *spa* and SCC*mec* types. The dominant *spa* types for ST152, ST22, ST30, ST5, ST6, ST672, ST8, ST88, and ST97 were t355, t005, t021, t311, t304, t3841, t008, t690, and t267, respectively (Figure 3). Except for ST97, the dominant *spa* subtypes in other clones contained genomes with the PVL genes. The diversity of SCC*mec* subtypes within each clone suggests pervasive dissemination, coexistence, and replacement of these subtypes. While some ST22 genomes carried SCC*mec* type I, commonly associated with HA-MRSA, the majority of SCC*mec* loci in the major clones were smaller, CA-MRSA-associated types IVa (*n* = 228), V/VII (*n* = 197), and VI (*n* = 17). This trend was more pronounced in dominant clones, with smaller SCC*mec* elements observed in ST8, ST88, ST6, and ST22 (Wilcoxon rank-sum test, *p* < 0.01), and slightly smaller elements in ST30 (*p* < 0.1) (Supplementary Figure S4). We also examined the association between the frequency of virulence and resistance gene content in major MRSA clones with different SCC*mec* types. As shown in Supplementary Table S3, subclones of ST8 and ST5 harboring SCC*mec* types V/VII, and IVa, respectively, contained a significantly higher number of virulence factor genes (one-sided Wilcoxon rank-sum test, *p* < 0.001) (Supplementary Table S3 Notably, the ST8-IVa subclone also carried a higher number of resistance genes, suggesting enhanced fitness associated with these lineages. In addition to ST8, ST22, and ST30 with SCC*mec* type IV elements also showed significantly increased counts of virulence and resistance genes (Supplementary Table S3). This suggests potential enhanced fitness and virulence associated with these lineages. Besides SCC*mec*-associated patterns, we also examined two highly conserved serine residues—S84 in *gyrA* and S80 in *parC*—within the QRDR of DNA gyrase and topoisomerase IV. These fluoroquinolone resistance mutations are not only known to confer resistance but have also been associated with fitness advantages linked with the acquisition of resistance and virulence genes (Fuzi et al., 2017, 2020). Our findings show that ST5 and ST8 MRSA carrying both mutations tended to have a higher virulence gene content, mirroring the pattern observed with SCC*mec* types (Supplementary Table S4). Similarly, ST5, ST8, and ST22 isolates with dual fluoroquinolone resistance mutations exhibited an elevated number of virulence genes. These observations suggest a potential fitness benefit associated with smaller SCC*mec* elements and QRDR mutations, which may contribute to the clonal expansion of these lineages, as previously suggested (Collins et al., 2010).

### Temporal origins and population dynamics of the clones

**Figure 4:**
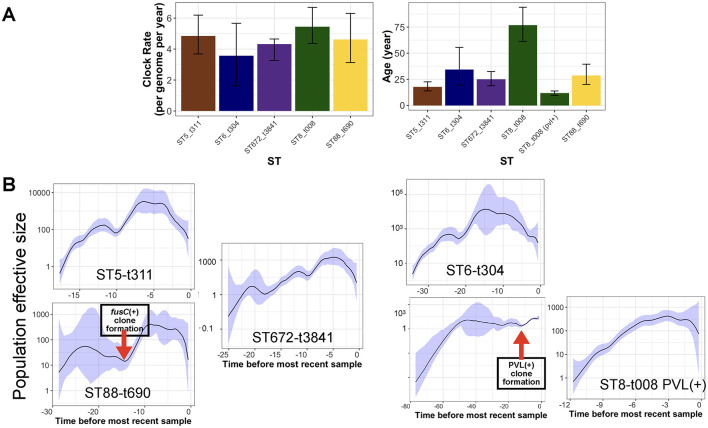
Phylodynamic analysis of clones with the most prevalent *spa* type. **(A)** The estimated clock rate and age values of the clones from Bayesian analysis. The error bars are the credible intervals [95% Highest Posterior Density (HPD)]. **(B)** The skyline growth for the clones in **(A)**, based on the Bayesian tree for the clones. The shaded area corresponds to 95% confidence interval.

**Figure 5:**
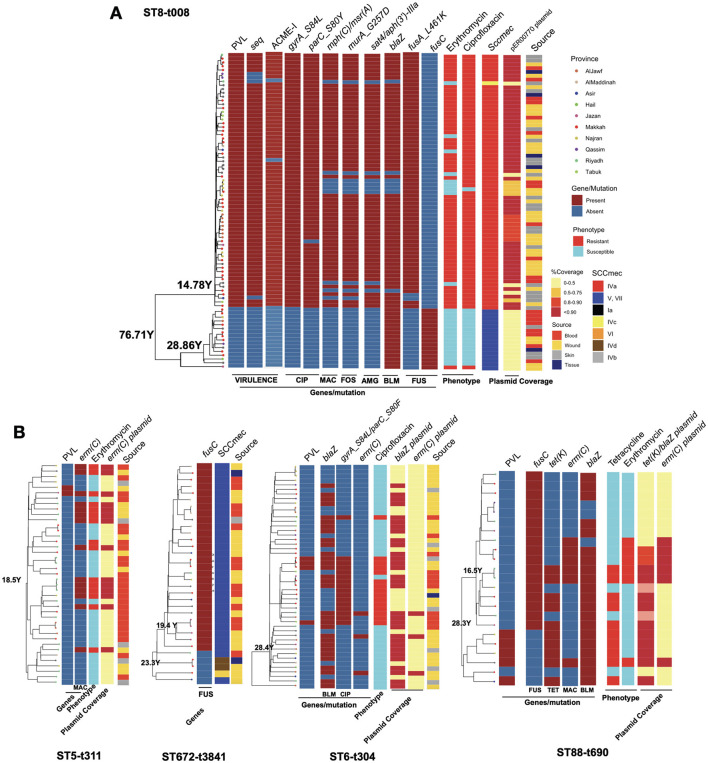
The phylogenetic trees for the clones with key resistance determinants, virulence factor genes, SCC*mec* type, antimicrobial resistance phenotype and the presence of key plasmids for the ST8-t008 clones in **(A)** and other expanding clones **(B)**. Only resistance determinants, virulence gene factors and antimicrobial resistance phenotypes that were variably present in each clone according to Figure 2, Supplementary Figures S1, S3 are shown. The plasmid band shows the coverage percentage after mapping the short reads against the plasmid with the resistance genes retrieved from long-read sequencing data. Color codes in **(B)** are the same as in **(A)**. The genomic map of the plasmids is provided in Supplementary Figure S2.

After integrating *spa* and ST typing, we identified five major clones representing over 20% of the total collection. These included ST8-t008 (USA300 clone), ST88-t690, ST672-t3841, ST6-t304, and ST5-t311. We utilized the temporal signal within these clones to analyze their population dynamics. The results indicate a comparable molecular clock across the clones, ranging from 3.2 to 4.5 substitutions per genome per year (Figure 4A). Despite the similar clock rates inferred from overlapping credible intervals, ST5-t311 and ST672-t3841 clones appear to have emerged more recently, with an estimated most recent common ancestor (MRCA) age of 20 to 30 years. ST8-t008 (USA300) clones were found to consist of two sub-clones, distinguished by the presence of PVL and resistance genes (Figure 5A). While the ST8-t008 lineage is long-standing (76 years), the PVL-positive subclade [ST8-t008 PVL(+)] emerged relatively recently, within the past 15 years, making it younger than the other clones (Figures 4A, 5A). All clones exhibited an overall increasing population trend over time, although a slight recent decline was observed in all clones (Figure 4B) (see Discussion). Although ST5 and ST6 had larger population sizes overall, ST5—together with ST8 following the emergence of its PVL-positive sub-clone—underwent the most rapid population expansion, increasing 1,000-fold over a ten-year period (Figure 4B). Similarly, the ST88-t690 clone experienced a rapid population increase 12 years ago, coinciding with the emergence of a *fusC*-positive clone (Figures 4B, 5B). Overall, the findings indicate comparable molecular clock rates, effective population sizes, and the recent emergence of the five clones, which could be linked to the acquisition of resistance/virulence determinants.

### Expanding clones revealed the emergence of new clones linked with the acquisition of antimicrobial resistance plasmids

We next analyzed the dynamics of the expanding clones and integrated this with resistance determinants and plasmids identified through third-generation sequencing. In the USA300 clone, the subclone carrying PVL genes exhibited a significantly different composition of antimicrobial resistance genes (Figure 5A). While isolates from both subclones were recovered from different body sites, the PVL(+) subclone harbored SCC*mec* type IVa, whereas the PVL(-) subclone carried type VI, which was linked to the *fusC* gene (Figure 5A). The PVL(+) subclone also contained other key virulence genes (e.g., the superantigen *seq* and ACME-I genes) and resistance determinants (e.g., resistance mutations for ciprofloxacin, fusidic acid, and fosfomycin, as well as resistance genes for aminoglycoside and macrolide). The presence of these genes was linked with the resistance to macrolides (erythromycin) and ciprofloxacin (Figure 5A). The macrolide and aminoglycoside resistance genes in the PVL(+) clones were located on a 27kb *blaZ*-containing plasmid (identical to the clinical pER00770_1 plasmid) (Supplementary Figure S2). Similar to the USA300 clone, other STs also exhibited mixing of isolates from different body sources, SCC*mec* replacement, the rise of clades with the PVL genes, and evidence of plasmid-borne resistance acquisition during clonal expansion (Figure 5B). The rise of PVL(+) lineages was observed in ST88-t690 and ST6-t304. Similar to the ST8 clade, ST672 contained two SCC*mec* types, with type V harboring the *fusC* gene, which emerged and expanded from a lineage with SCC*mec* IVd. The *fusC* gene exhibited distinct genomic contexts in the ST672 and ST88 clades: in ST672, it was located separately from SCCmec, whereas in ST88, it was integrated within a chimeric SCC*mec*–SCC*fus* element, which has not been widely reported for this ST. The ST6-t304 and ST88-t690 clades acquired *blaZ*-containing plasmids with similar replicons, with the ST88-t690 clone also carried the *tet(K)* gene (Figure 5B, Supplementary Figure S2). The acquisition of these plasmids was linked to resistance to macrolides and tetracycline. Moreover, our results show the sharing of a 2.6kb *emr(C)*-containing plasmid (Supplementary Figure S2) among subclades within ST5-t311, ST6-t304, and ST88-t690 clones (Figure 5B).

### CA-MRSA clones spread across the hospitals within the country

We then examined the spread of the five clones across hospitals by reconstructing the transmission network for each clone, assuming that ancestor and descendant genomes were sampled concurrently. 22 SNP cut-off, which corresponds to hospital transmission over a 6-month period (see Methods), we identified 41 transmission networks for the five abovementioned expanding clones, accounting for 15%−28% of the isolates within each clone (Supplementary Figure S5). The largest proportion of isolates involved in a transmission network was observed for the ST8-t008 clone, which, except for the ST88-t690 clone, was higher than the other clones. The transmission links spanned all regions of the country for all clones (Supplementary Figure S5). The ST8-t008, ST672-t3841, and ST88-t690 strains showed recent genetic relatedness with European clinical samples, each forming part of three SNP clusters in the Pathogen Detection database. ST88-t690 clustered with Danish clinical isolates at a closest SNP distance of 37 (SNP cluster PDS000144949.9), which, although above the typical transmission threshold, still suggests cross-border dissemination. Similarly, ST672-t3841 grouped with Dutch clinical samples at an SNP distance of 11 (SNP cluster PDS000170231.2), indicating the spread of this clone beyond Saudi Arabia.

### Plasmidome analysis indicates diversity and horizontal transfer of *blaZ* containing plasmids

Our analysis of *blaZ*-containing plasmids, retrieved from long-read sequencing data, revealed significant plasmid diversity, which could be grouped into ten plasmid clusters based on their origins of replication (Table 1) (see Methods). The large diversity of replicons is also evident in the wide variation in plasmid lengths, which ranged from 12 Kb to over 42 Kb, with larger plasmids generally carrying a greater number of resistance genes (Table 1). These resistance plasmids exhibited various combinations of resistance genes significantly associated with resistance phenotypes, including *blaZ, blaI, blaR1, cadD, tet(K), and aac(6')-Ie/aph(2”)-Ia*, leading to the formation of multidrug-resistant plasmids. Except for one plasmid cluster, all clusters contained *cadCD* heavy metal resistance genes. The plasmids were distributed across different cities and body sites within five clusters. All plasmid clusters harbored *OriT* regions, as demonstrated for the *blaZ*-containing plasmids in the major clones in Supplementary Figure S2, which enables plasmid transfers (Ramsay et al., 2016; Kwong et al., 2017). Furthermore, a similar collection of plasmids was observed for five plasmid clusters of different ST types, suggesting the dissemination and sharing of plasmids across diverse genetic backgrounds (Table 1). The widespread distribution of plasmids across bacterial hosts and locations points to extensive plasmid transmission within hospital settings, likely facilitated by human carriers or hospital environments (David and Daum, 2010; Harris et al., 2010).

**Table 1:** List of *blaZ*-containing plasmids retrieved from long-read sequencing data.

**Plasmid cluster**	**Size of cluster**	**Length range (bp)**	**Resistance genes**	**Antmicrobial class**	**STs**	**City**	**Sites**
1	1	22K	*blaI, blaR1, blaZ, cadD*	BLA, HMT	ST1930	Riyadh	Blood
2	3	20K	*blaZ, blaR1, blaI, cadD*	BLA, HMT	ST672, ST291	Jazan, Najran	Blood, Wound
3	1	13K	*blaZ, blaR1, blaI, msr(A), cadD*	BLA, HMT, MAC	ST834	Algryat	Skin
4	1	26K	*cadD, ant(6)-Ia, sat4, aph(3')-IIIa, tet(K), blaI, blaR1, blaZ*	AMG, BLA, HMT, TET	ST789	Taif	Wound
5	15	19K,-20K	*cadD, blaI, blaR1, blaZ*	BLA, HMT	ST6, ST1153, ST152, ST97, ST9337	Riyadh, Dammam, AlQassim, Jeddah, Hail	Skin, Tissue, Wound, Blood
6	2	27K	*cadD, blaZ, blaR1, blaI, mph(C), msr(A), ant(6)-Ia, sat4, aph(3')-IIIa*	AMG, BLA, HMT, MAC	ST1	Hail	Skin
7	5	24K-25K	*cadD, msr(A), blaI, blaR1, blaZ, fusB, bleO, aadD1, mph©, ant(6)-Ia, sat4, aph(3')-IIIa*	AMG, BLA, BLE, FUS, HMT, MAC	ST2867, ST1482, ST8	Najran, Dammam, Algryat, AlQassim	Blood, Skin
8	3	28K-29K	*cadD, tet(K), blaI, blaR1, blaZ, tet(K), fusB*	BLA, FUS, HMT, TET	ST80, ST88	Jeddah, Najran	Blood, Wound
9	2	23K	*fusB, blaZ, blaR1, blaI, cadC*	BLA, FUS, HMT	ST997, ST80	Hail	Skin, Blood
10	5	33K, 42K	*lnu(A), aadD1, tet(K), blaI, blaR1, blaZ, msr(A), mph(C), bleO, fusB*	AMG, BLA, BLE, FUS, LIN, MAC, TET	ST1535, ST9337, ST15, ST30, ST88	Dammam, Riyadh	Blood, Tissue, Wound

Each group corresponds to plasmids with a unique origin of replication. The order of antimicrobial classes corresponds to the order of genes in the resistance genes column. The annotations for the abbreviations are as follows: BLA, β-lactam antimicrobials; HMT, Heavy metal tolerance; MAC, Macrolides; AMG, Aminoglycosides; TET, Tetracyclines; BLE, Bleomycin resistance; FUS, Fusidic acid; LIN, Lincosamides. The size of the cluster indicates the number of plasmids in the cluster.

## Discussion

### Summary

We conducted a large-scale genomic epidemiology analysis of a *S. aureus* collection, mostly comprised MRSA strains, retrieved from a large hospital network across the Kingdom of Saudi Arabia. Utilizing a range of genomic epidemiology techniques, we described the population diversity, identified novel MRSA strains, and examined the recent evolution of emerging and expanding clones in the hospitals in Saudi Arabia. Strains exhibited specific infection sites, reflecting differences in pathogenicity or ecological adaptation, likely driven by specific virulence genes. By employing a hybrid sequencing approach, we uncovered extensive diversity and sharing of AMR-linked plasmids between clones. The findings also revealed significant within-clone diversity and recent evolutionary events, including the concurrent acquisition of plasmid and clonal expansion. Given the diversity and breadth of the hospitals included in this study, we expect the findings to be generalizable to the broader Middle East and West Asia region.

### Regional variation in MRSA prevalence

We found no association between human population size and MRSA prevalence in our nationwide analysis across provinces. This lack of correlation suggests that other factors—such as differences in healthcare infrastructure, antimicrobial usage, and population mobility—likely play a more significant role in shaping MRSA prevalence, as reported by previous studies (Köck et al., 2010; Cameron et al., 2019). A previous systematic review in Saudi Arabia also reported regional variation in MRSA prevalence, with higher rates in the Western region (42%) compared to the Central (32%) and Eastern (27%) regions. The elevated prevalence in the Western region is likely attributable to higher population mobility and specific healthcare practices in that area (Aljeldah, 2020). These findings highlight the need for region-specific data to inform targeted interventions.

### Population diversity of MRSA in Saudi Arabia reflects the regional diversity

The study revealed a genetically diverse population of co-circulating MRSA lineages in Saudi Arabia, carrying a wide array of genetic determinants linked to antimicrobial resistance and virulence. The dominant clones identified were either pandemic or regionally significant. ST8-t008 (USA300), the predominant clone, is a well-known pandemic CA-MRSA lineage, particularly prevalent in North America but also reported in other regions, including the Middle East (Boswihi et al., 2016). Similarly, ST6-t304, part of the CC6 CA-MRSA lineage, has demonstrated the ability to spread internationally, especially in Northern Europe (Avison et al., 2002; Venla et al., 2023). Other major clones identified have been reported in Africa, East Asia, and the Middle East: ST88-t690 belongs to the ST88-IV African CA-MRSA clone (Harris et al., 2010), while ST672-t3841 is part of the CC361 lineage, and ST5-t311 is part of the CC5 clone, prevalent in the Middle East and South and East Asia (Sarkhoo et al., 2021; Alkuraythi et al., 2023; Murugadas et al., 2017). At the regional level, only a limited number of studies have investigated the prevalence of MRSA strains using a multicenter approach across GCC countries, and most of these have relied on non-WGS-based methodologies. The most recent and robust data indicate that ST22 is the dominant MRSA sequence type among clinical isolates in Kuwait (Boswihi et al., 2020, 2018), and it is also highly prevalent in the UAE (Senok et al., 2020). Other clones, including ST5, ST8, and livestock-associated lineages such as ST97, are present at lower yet still notable frequencies. Another recent multicenter study from the UAE identified ST5, ST6, ST22, and ST8 as the predominant clones (Boucherabine et al., 2025). In Oman, single-center data indicate ST6-IV-t304 as the predominant clone, which also appears as a prevalent clone in our collection (Udo et al., 2014). Other common clones such as ST22, ST5, ST30, and ST8 were less frequent in their cohort. Although ST152 has not been widely reported in the GCC region, but its incidence in Africa suggests its emergence in Saudi Arabia is a result of introduction from Africa (Egyir et al., 2024, 2021). The shared dominant clones across countries suggest regional circulation driven by human movement and mass gatherings, highlighting the need for a unified genomic study to understand inter-country transmission.

### Regional consistency and emerging genomic features in Saudi MRSA strains

In addition to shared sequence types (STs), our collection exhibited other genomic features consistent with previous reports on MRSA in Saudi Arabia and the region. For instance, our results indicate a complete absence of *mecC*, aligning with findings from other regional studies reporting either complete absence in Turkey (Cikman et al., 2019) or low prevalence in Iran (Goudarzi et al., 2020). Some findings indicate the emergence of novel pathogenic and resistance features in Saudi Arabia. Notably, the rise of ST88-t690-SCCmecIVa with chimeric SCC*mec*–SCC*fus* elements, and the detection of ACME-I in rare lineages beyond ST8, suggest ongoing genomic diversification and local emergence of clinically significant MRSA strains.

### USA300 clone in Saudi shows similarity to the prevalent clone in the Americas

The ST8-t008 clone in our collection is predominantly composed of the USA300 variant, displaying all the hallmark characteristics observed in US hospitals, including SCC*mec* type IV, the *S. aureus* pathogenicity island 5 (SaPI5), and a multidrug-resistant 27 Kb plasmid (Diep et al., 2006; Kennedy et al., 2010). The formation dates for both the ST8-t008 and USA300 clones align with the reported ages of these lineages in the Western Hemisphere (Planet et al., 2015; Smith et al., 2022; Souza et al., 2024), suggesting that the expanding clone in Saudi Arabia mirrors the global expansion of the ST8 lineage, particularly the USA300 strains. The minor PVL(-) sub-clone of ST8-t008 with SCC*mec* V in our collection is less well-documented and was reported as a rare strain in a large-scale, single-hospital study in the US (Souza et al., 2024). The spread and replacement of accessory resistance and virulence genes, including SCC*mec* subtypes was also reported during hospital outbreaks and inter-hospital transmissions (Weterings et al., 2017). This is likely driven by the higher fitness of smaller SCC*mec* subtypes, along with the co-selection and mobility of resistance genes (Collins et al., 2010; Smith et al., 2022). The coexistence of ST8-t008 sub-clones in our population may represent a snapshot of the population dynamics, where a fitter clone/sub-clone dominates and outcompetes others. Similar to previous studies, we observed a recent decline in population size of the clones, which has been previously attributed to improved infection control efforts in hospitals or the emergence of newly successful but rare clones (Kourtis et al., 2019). To determine which factor underlies this dynamic pattern, a long-term longitudinal study incorporating epidemiological data, genomic surveillance, and transmission modeling would be necessary.

### Limitation and the need for One Health framework

Despite the breadth of our study, there are several limitations. Firstly, we lacked detailed clinical data, such as patient infection sources, travel history, infection types, and clinical outcomes. This limitation restricted our ability to draw conclusions about the clinical significance of emerging clones. Human mobility, particularly international travel, as it is common in Saudi Arabia, plays a crucial role in the introduction and dissemination of CA-MRSA strains, facilitating the spread of diverse clones within communities and healthcare settings (Boswihi et al., 2018; Senok et al., 2020). Including information such as travel history in the analysis could help decipher the factors contributing to the spread of the clones across provinces. Secondly, the absence of environmental samples in our study did not allow us to determine the role of non-human reservoirs and agents in disseminating the strains across hospitals. Among the dominant clones, the only STs previously reported in animal hosts in Saudi Arabia and the broader GCC region are ST6, ST5, ST97, and ST672—primarily isolated from goats or retail meat (Alkuraythi et al., 2023; El-Deeb et al., 2022, 2018; Raji et al., 2016). Most animal-associated STs exhibited sequence similarity to local human clinical isolates, suggesting ongoing transmission between reservoirs (Alkuraythi et al., 2023; El-Deeb et al., 2022, 2018; Raji et al., 2016). This potential overlap underscores the need for large-scale, systematic, and unified studies that integrate human, animal, and environmental reservoirs in a One-Health framework.

## Conclusion

Our study underscores the importance of understanding the regional epidemiological characteristics of MRSA clones in a highly diverse geographical setting, which remains significantly underrepresented in global pathogen genomic databases. Despite its limitations, this dataset offers a vital baseline of the genetic diversity and antimicrobial resistance of *S. aureus* in the Middle East, enhancing understanding of local MRSA patterns and supporting global surveillance of high-risk clones.

## Data Availability

The datasets presented in this study can be found in online repositories. The names of the repository/repositories and accession number(s) can be found in the article/Supplementary material.
