# Supplementary material for: Methicillin-resistant Staphylococcus aureus in Saudi Arabia: genomic evidence of recent clonal expansion and plasmid-driven resistance dissemination
Source: Front Microbiol. 2025 Jun 13;16:1602985. doi: 10.3389/fmicb.2025.1602985 (PMC12202538; doi:10.3389/fmicb.2025.1602985)
Supplement: Supplementary file 2 [file Data_Sheet_1.pdf]

Supplementary Figures

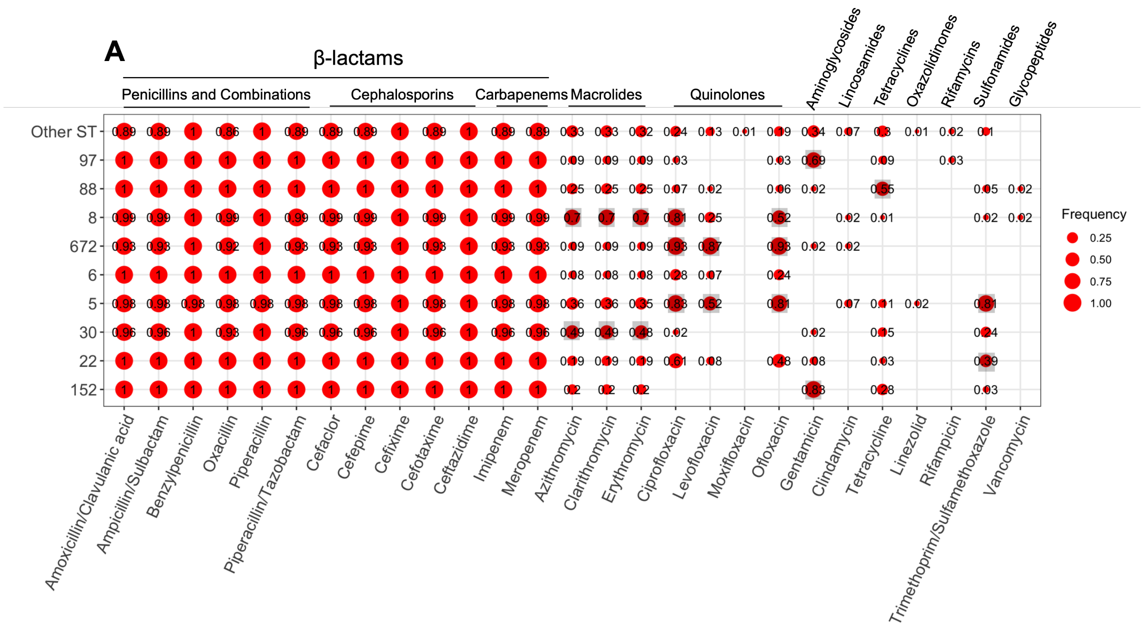

Figure S1: The frequency of resistance phenotypes across major STs. The grey squares indicate resistance levels that were significantly higher in each ST compared to the rest of the collection (p-value < 0.01 from a one-sided proportion test).

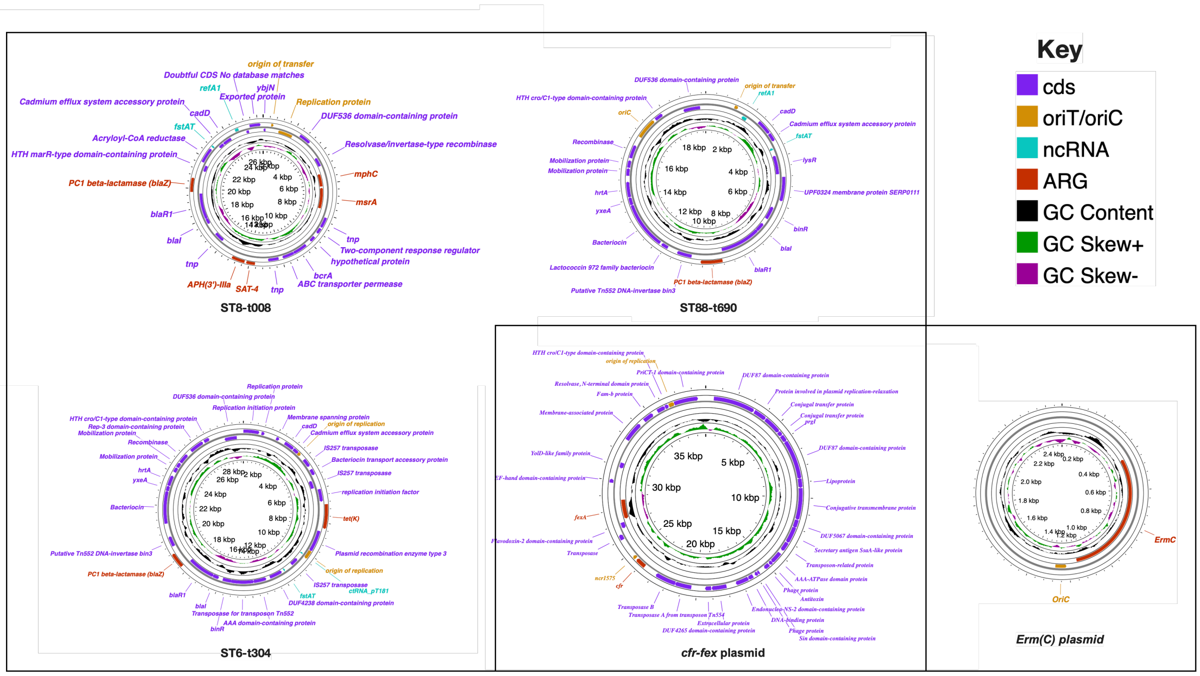

Figure S2 The genomic map of the plasmids shown in Figure 6 and mntioned throughout the text. The antimicrobial resistance genes (ARGs) were reported according to CARD. Figures were generated by www.proksee.ca built-in tools including annotation and ARG finding pipelines.

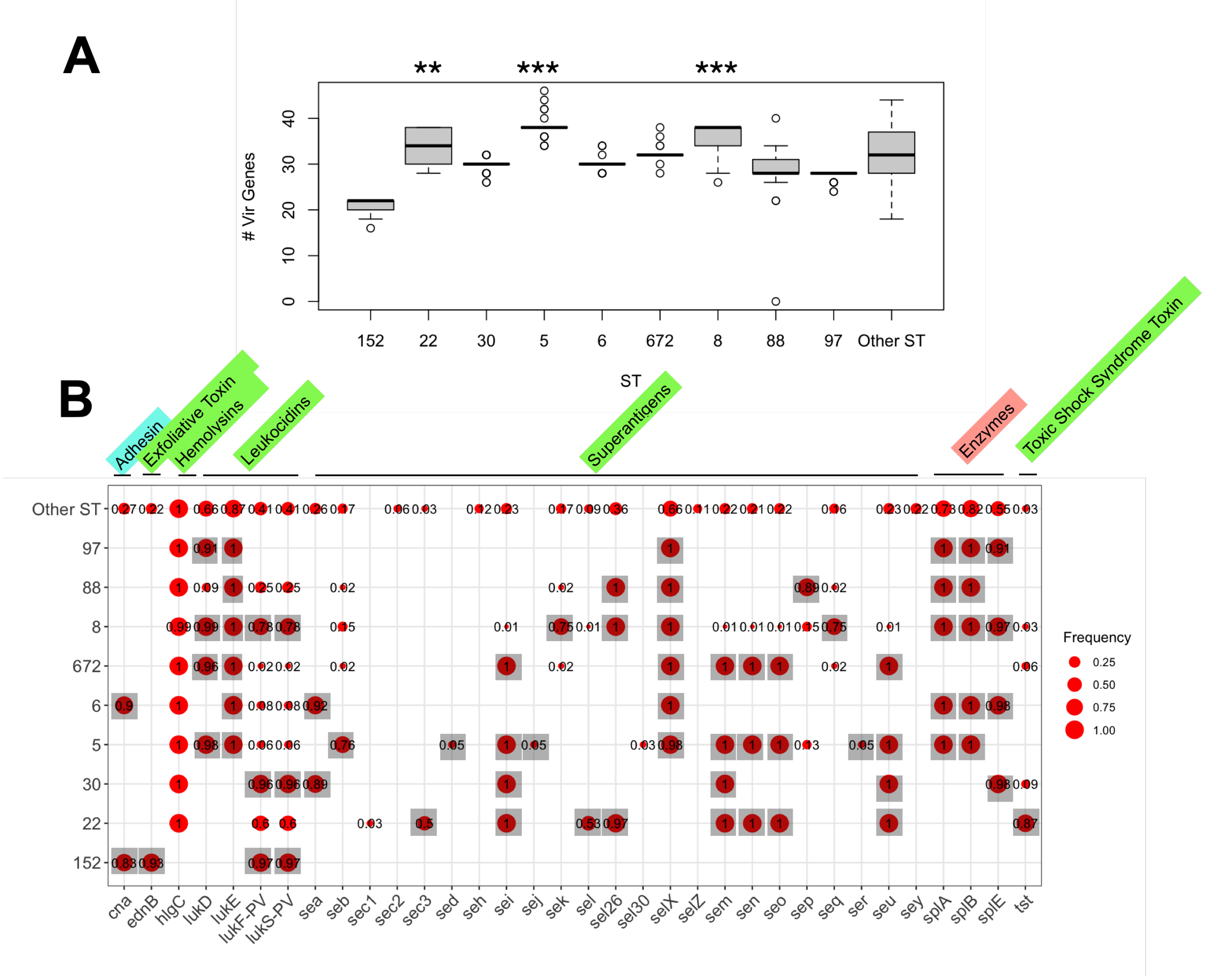

Figure S3 A) The distribution of virulence factors across the major ST clones. Each boxplot denotes the count of resistance determinanats or viruelnce factor genes idenfitied srst2 tools (using VFDB), respectively. The ** and *** signs corresponds to the significance levels of <0.01 and <0.001, respectively, from the one-sided proportion test, indicating whether the mean frequsny of viruelnce factor genes count were higher in the ST clone compared to the rest of the collection. B) The frequency of virulence factor genes across the major STs. The grey squares shows genes/mutations/drugs which had a siginficantly higher frequency in each ST compared to the rest of the collection (p-value<0.01 from one-sided proportion test).

Table S2. Missense polymorphisms in the PVL genes *lukS* and *lukF,* using *Staphylococcus aureus* subsp. aureus MW2 (NC_003923), an ST8 strain, as the reference genome.

**ST**	***lukF-PV***		***lukS-PV***	
	**Tyr304Phe**	**Glu322Lys**	**Arg176His**	**Phe157Tyr**
152			1	24
22			19	8
30		30	40	33
5			2	
6			1	
672			1	
8	3			
88			14	8

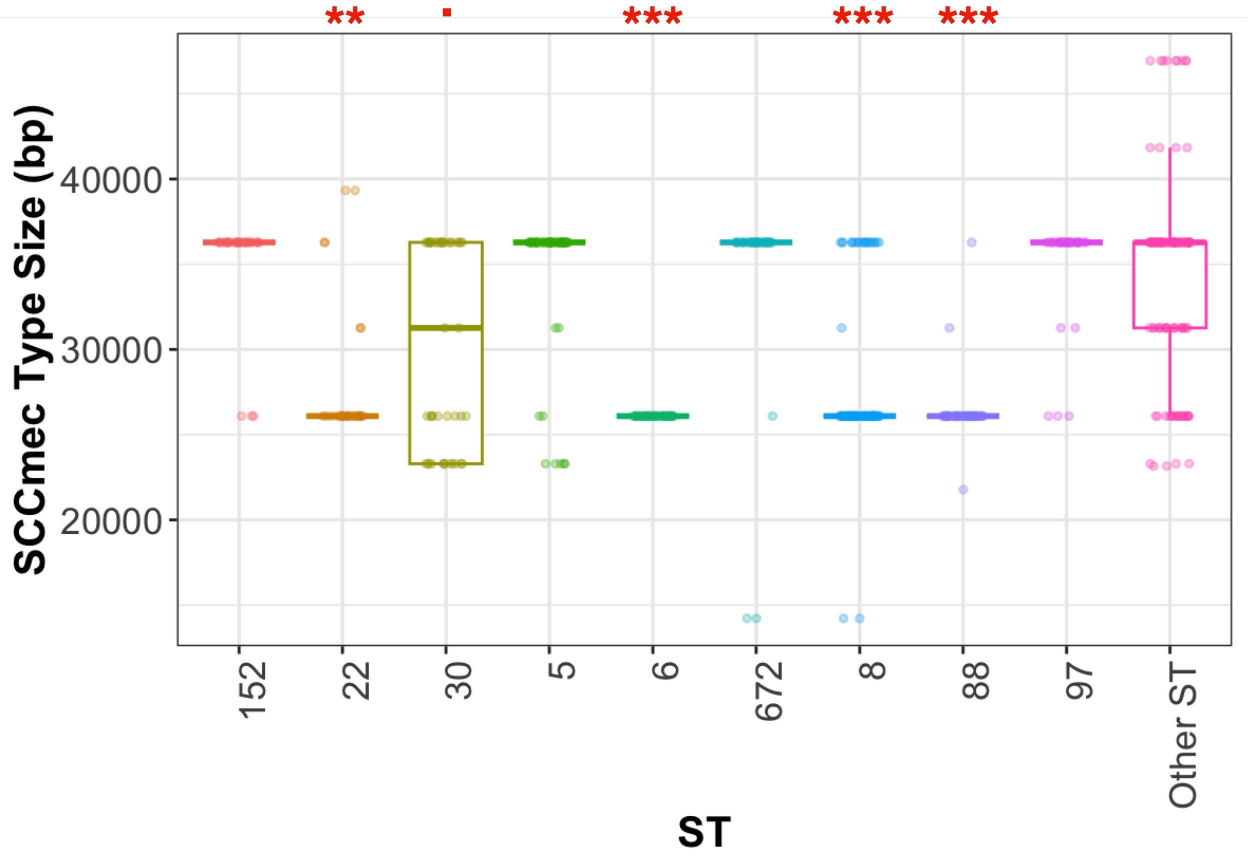

Figure S4 Distribution of SCC*mec* element sizes across major MRSA clones. Each dot represents the SCC*mec* size of a single genome. The symbols (.), (**), and (***) indicate significance levels of $p < 0.1$, $p < 0.01$, and $p < 0.001$, respectively, based on one-sided Wilcoxon rank-sum tests assessing whether a given clone has significantly smaller SCC*mec* elements compared to the rest of the population.

Table S3: Frequency of resistance and virulence genes among isolates in major clones with different SCC*mec* types, as determined by the staphopia-sccmec pipeline. The second column reports the number of isolates within each ST clone carrying different SCC*mec* element. The resistance and virulence gene count columns show the average, minimum, and maximum number of genes detected by the AMRFinderPlus pipeline. Sign # denotes the count. Gene groups with significantly higher gene counts than the rest of the population are highlighted in bold red. Significance levels from one-sided Wilcoxon rank-sum tests are indicated by (*) and (***) corresponding to p < 0.01, and p < 0.001, respectively.

ST	Size	SCC*mec* type/subtype	#SCC*mec*	# Virulence Genes (Mean, minimum, maximum)	# Resistance Genes (Mean, minimum, maximum)
5	64	IVa	2	18.5 (18,19)	9.5 (9,10)
		IVc	2	**20.5 (18,23) ***	8 (8,8)
		V, VII	54	**19.03 (17,22) *****	10.39 (7,16)
		VI	5	**18.6 (17,19) ****	11.4 (9,21)
6	54	IVa	54	14.96 (14,17)	7.68 (5,11)
8	110	IVa	85	**18.67 (17,19) *****	**16.43 (9,18) *****
		IVc	1	18	9
		IVd	2	16 (16,16)	13 (9,17)
		V, VII	21	16.19 (13,17)	7.28 (7,11)
22	31	Ia	2	15 (15,15)	6 (6,6)
		IVa	25	**17.8 (14,26) *****	10.04 (7,12)
		IVc	2	16 (16,16)	13.5 (13,14)
		V, VII	2	15 (15,15)	6 (6,6)
30	46	IVa	9	14.11 (13,15)	**14.22 (12,20) *****
		IVc	2	14 (14,14)	12 (12,12)
		V, VII	21	15 (15,15)	**13.43 (10,14) *****
		VI	12	15.33 (15,16)	**15.75 (13,18) *****
88	56	IVa	53	14.28 (11,16)	8.60 (5,11)
		IVb	1	17	9
		IVc	1	17	13
		V, VII	1	20	13
97	35	IVa	3	13 (13,13)	8 (8,8)
		IVc	2	14 (14,14)	7 (7,7)
		V, VII	30	13.76 (12,14)	7.96 (4,9)

152	30	IVa	3	10.66 (10,11)	10.66 (10,11)
		V, VII	27	10.63 (8,11)	**11.60 (11,13) ***
672	46	IVa	1	19	10
		IVd	2	16	11
		V, VII	40	16.02(14,18)	11.05 (8,16)

Table S4: Frequency of resistance and virulence genes among isolates in major clones with different combinations of double-serine QRDR mutations (*gyrA* S84L and *parC* S80F/Y). The binary indicators (0 and 1) denote the presence and absence of the *gyrA* S84L and *parC* S80F/Y mutations. The third column reports the number of isolates within each ST clone carrying the mutation combination. The resistance and virulence gene count columns show the average, minimum, and maximum number of genes detected by the AMRFinderPlus pipeline. Sign # denotes the count. Gene groups with significantly higher gene counts than the rest of the population are highlighted in bold red. Significance levels from one-sided Wilcoxon rank-sum tests are indicated by (*) and (***) corresponding to p < 0.01, and p < 0.001, respectively.

ST	Size	Fluroquinolone *gyrA* S84L and *parC* S80F/Y	# Fluroquinolone	# Virulence factor Genes (Mean, minimum, maximum)	# Resistance Genes (Mean, minimum, maximum)
5	64	0/0	11	**19.45 (18,23) *****	7.81 (3,10)
		1/0	0		
		0/1	0		
		1/1	53	**18.94 (17,22) *****	10.75 (9,21)
6	54	0/0	36	14.77 (14,15)	6.97 (5,9)
		1/0	0		
		0/1	1	15	9
		1/1	17	15.35 (14,17)	9.11 (7,11)
8	110	0/0	21	16.23 (13,18)	7.19 (7,9)
		1/0	1	19	16
		0/1	0		
		1/1	88	**18.56 (16,19) *****	**16.19 (7,18) *****
22	31	0/0	13	15.92 (14,26)	7.38 (6,12)
		1/0	0		
		0/1	0		
		1/1	18	**18.33 (16,19) *****	**11.44 (11,14) ***
30	46	0/0	45	14.88 (13,16)	**13.88 (10,18) *****
		1/0	0		
		0/1	0		
		1/1	1	14	20
88	56	0/0	52	14.26 (11,17)	8.63 (5,13)
		1/0	0		
		0/1	0		
		1/1	4	17.25 (16,20)	10.5 (9,13)
97	35	0/0	32	13.75 (12,14)	7.90 (4,9)
		1/0	0		
		0/1	1	12	9
		1/1	2	14	7.5 (7,8)
152	30	0/0	30	10.63 (8,11)	**11.5 (10,13) *****
		1/0	0		
		0/1	0		
		1/1	0		

672	46	0/0	2	16	6
		1/0	0		
		0/1	0		
		1/1	44	16.09 (14,19)	11 (8,16)

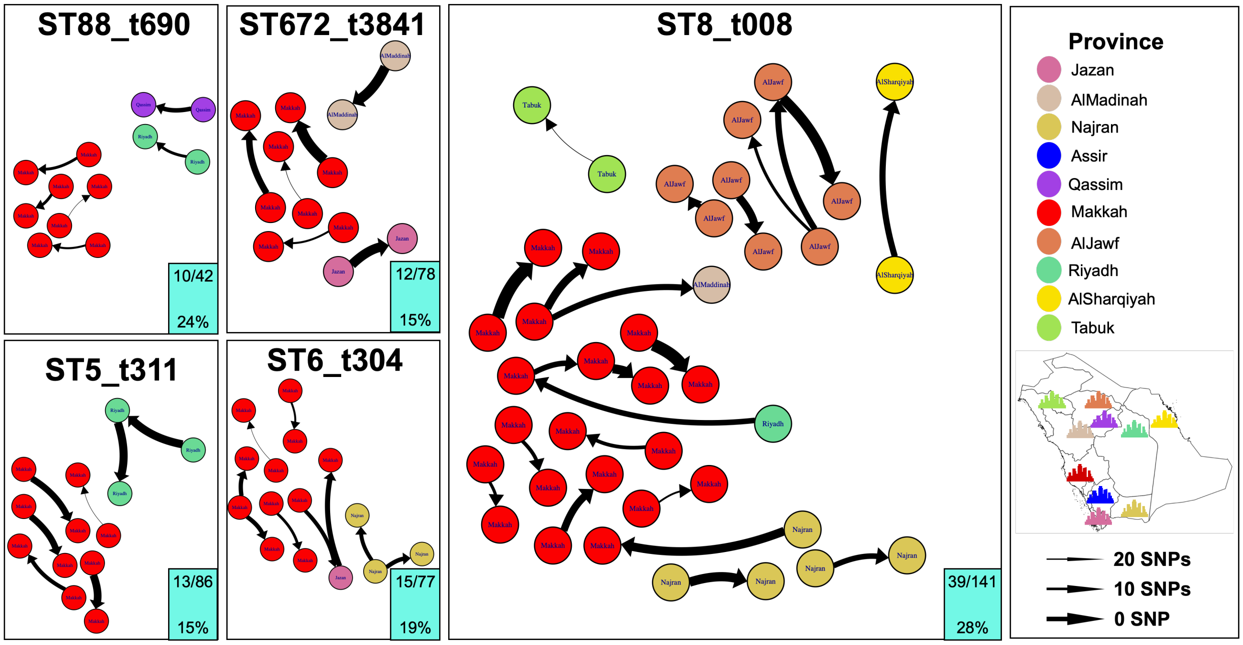

Figure S5 The transmission network analysis for the identfied recent clones. The nodes and edges denote the isolates and transmission network, respectively. Colors irepresent provinces. The thickness of the edges denote the transmission link.
